# Development of precision of non-symbolic arithmetic operations in 4-6-year-old children

**DOI:** 10.3389/fpsyg.2023.1286195

**Published:** 2023-11-15

**Authors:** Chen Cheng, Melissa M. Kibbe

**Affiliations:** ^1^Division of Social Science, School of Humanities and Social Science, The Hong Kong University of Science and Technology, Hong Kong, Hong Kong SAR, China; ^2^Department of Psychological & Brain Sciences, Boston University, Boston, MA, United States

**Keywords:** non-symbolic arithmetic, development, approximate number system, addition, solving for unknown-addend, precision

## Abstract

Children can represent the approximate quantity of sets of items using the Approximate Number System (ANS), and can perform arithmetic-like operations over ANS representations. Previous work has shown that the representational precision of the ANS develops substantially during childhood. However, less is known about the development of the *operational* precision of the ANS. We examined developmental change in the precision of the solutions to two non-symbolic arithmetic operations in 4-6-year-old U.S. children. We asked children to represent the quantity of an occluded set (Baseline condition), to compute the sum of two sequentially occluded arrays (Addition condition), or to infer the quantity of an addend after observing an initial array and then the array incremented by the unknown addend (Unknown-addend condition). We measured the precision of the solutions of these operations by asking children to compare their solutions to visible arrays, manipulating the ratio between the true quantity of the solution and the comparison array. We found that the precision of ANS representations that were not the result of operations (in the Baseline condition) was higher than the precision of solutions to ANS operations (in the Addition and Unknown-addend conditions). Further, we found that precision in the Baseline and Addition conditions improved significantly between 4 and 6 years, while precision in the Unknown-Addend condition did not. Our results suggest that ANS operations may inject “noise” into the representations they operate over, and that the development of the precision of different operations may follow different trajectories in childhood.

## 1. Introduction

Humans can rapidly estimate the approximate quantity of sets of items without counting or relying on symbolic notation. This ability is supported by the Approximate Number System (ANS; Dehaene, 1997; Feigenson et al., 2004), a core cognitive system that is operational in infants and young children before they begin receiving formal mathematics training (Gallistel and Gelman, 1992; Xu and Spelke, 2000; Lipton and Spelke, 2003; Barth et al., 2005, 2006). The ANS allows us to approximately quantify sets of objects by representing these quantities as noisy magnitudes (Meck and Church, 1983; Gallistel and Gelman, 1992), and the ability to discriminate these magnitudes depends both on the size of the quantities that they represent and the extent of the difference between the quantities (Lipton and Spelke, 2003; Roitman et al., 2007; Prather, 2012, 2014; DeWind et al., 2015).

Previous work has shown that the representational precision of the ANS develops significantly across the lifespan, from infancy into late adulthood (Xu and Spelke, 2000; Lipton and Spelke, 2003; Halberda and Feigenson, 2008; Halberda et al., 2012; see Odic and Starr, 2018, for review) and precision can vary across individuals (Libertus et al., 2013; Thompson et al., 2016; Prather, 2019). In many of these studies, participants are asked to compare the relative magnitudes of visible arrays (e.g., participants are shown two sets of dots and are asked which set has more dots). Using this method, Halberda and Feigenson (2008) found that 3-year-old U.S. children could discriminate two sets of dots with a ratio between their quantities of 2:3; by age 5, children successfully discriminated two quantities with a ratio of 4:5, and by age 6 children could discriminate two quantities with a ratio of 6:7 between their quantities. ANS precision appears to develop independently from other magnitude representational capacities, including area, length, and time (Odic, 2018).

Individuals also can perform *operations* on ANS representations, transforming or manipulating ANS representations in the face of real-world changes to quantities, and this ability is already present in infancy and childhood (McCrink and Wynn, 2004; Barth et al., 2006; Booth and Siegler, 2008; McCrink and Spelke, 2010, 2016; Kibbe and Feigenson, 2015, 2017; Qu et al., 2021; Szkudlarek et al., 2022). For example, children who observe a set of dots move behind an occluder, and then observe a second set of dots move behind the same occluder, can update their representation of the quantity behind the occluder, effectively summing over their ANS representations of the sets (Barth et al., 2006). Further, Kibbe and Feigenson (2015, 2017) found that U.S. children could “solve for x” in non-symbolic unknown addend problems: children who observe a set of items which is then occluded, and then revealed to have increased in quantity, can compute the difference between the final quantity and initial quantity to infer approximately how many items were added, and can discriminate this solution from other smaller or larger quantities (see also Cheng and Kibbe, 2023). These studies suggest that ANS representations can be manipulated without direct visual access to the quantities they represent (e.g., two sequentially hidden sets can be combined to yield a representation of their sum, even when the combined set is never visible to children). Further, these studies show that the outputs of ANS operations are themselves ANS representations, which can then be compared to other ANS representations.

While much research has examined developmental change in the *representational* precision of the ANS, less is known about how the “operational precision” of the ANS may develop. To what extent does performing operations over ANS representations impact the precision of the outputs of these operations, and how might that change with development? ANS operational precision is likely to be at least somewhat dependent on the precision of the representations over which the operations are performed. Consistent with this, previous work showed that 5-year-olds who were asked to compute the sum of two sequentially-presented arrays were able to discriminate the summed total from a comparison array with ratios between the summed quantity and comparison array up to 4:5 (Barth et al., 2005, 2008; Gilmore et al., 2007) similar to the estimates of representational precision in this age group (e.g., Halberda and Feigenson, 2008). This suggests the possibility that the operational precision of the ANS may follow a similar developmental trajectory to the representational precision of the ANS. However, it is also possible that the process of actively manipulating ANS representations may result in different levels of noise injected into the outputs of ANS operations, and therefore that different operations may yield differentially noisy results. Consistent with this, Barth et al. (2006) found that 5-year-old children performed better on a non-symbolic addition task (two arrays sequentially occluded) compared to a non-symbolic subtraction task (one array occluded, followed by the removal of a subset of the items) (see also Barth et al., 2005, 2008; McCrink et al., 2007; Jang and Cho, 2022). If different ANS operations result in differential precision of the outputs of these operations, then it is possible that the precision of different ANS operations may follow different developmental trajectories as well. It is also possible that children may become more adept at some non-symbolic operations over time.

Previous work that examined the precision of the outputs of ANS operations in children did not include age as a factor in their designs, so the development of ANS operational precision – and whether and to what extent the precision of different ANS operations follows the same developmental trajectory as ANS representational precision – remains unknown. The goal of the present study was to examine the developmental trajectory of the precision of different ANS operations between the ages of 4 and 6 years, a period of substantial development in ANS representational precision (see, e.g., Halberda and Feigenson, 2008). In a within-participants design, children completed blocks of trials in which they were asked to solve different types of non-symbolic arithmetic problems over sets of dots, and then to compare their representation of the solution to a visible comparison array.

Because we used a within-participants design involving a high number of trials, we had to be strategic about which operations we investigated in order to explore ANS operational development while also minimizing task fatigue in our young participants. We therefore focused on two non-symbolic operations: addition (e.g., Barth et al., 2006) and solving for an unknown addend (e.g., Kibbe and Feigenson, 2015). In the Addition condition, children were asked to estimate the summed total of two arrays of dots that were shown and then hidden behind an occluder one at a time (similar to, e.g., Barth et al., 2006), and to then compare their representation of the total to a visible comparison array. In the Unknown-addend condition, children were asked to estimate an unknown added value after viewing an array of dots which was then occluded and revealed to have been incremented by some value, and then compare that estimate to a visible comparison array. We also included a Baseline condition, in which children were shown an array which was then occluded, and were then asked to compare their representation of the occluded array to a visible array. The Baseline condition allowed us to measure children’s representational precision for occluded sets, which provided a fairer comparison to the Addition and Unknown-addend conditions than measuring precision for visible sets (as in previous work, e.g., Halberda and Feigenson, 2008). We manipulated the ratio between the ANS representation stored in working memory (the hidden array in the Baseline condition, the summed total in the Addition condition, or the solved addend in the Unknown-addend condition) and the visible comparison array in order to explore the precision of these operations across development.

We chose to test addition and unknown-addend operations for several reasons. First, previous work has shown that children as young as 4 years old can solve both non-symbolic addition and unknown-addend problems (e.g., Barth et al., 2006; Hyde et al., 2014; Kibbe and Feigenson, 2015, 2017; Cheng and Kibbe, 2023), making these operations appropriate for our population of interest. Second, these operations may not be executed in the same way (see Cheng and Kibbe, 2023). In the Addition condition, children had to hold one ANS representation in working memory, and then update that representation by incrementing it by the new quantity, finally holding the resulting updated ANS representation in working memory. In the Unknown-addend condition, children had to hold one ANS representation in working memory, observe the new quantity after the unknown quantity was added, and then estimate the quantity of that unknown addend by computing the difference between the ANS representation held in working memory and the final quantity. Thus, both operations require children to store ANS representations in working memory, but require different operations to perform, making them good comparison cases.

Third, while previous work has demonstrated that 4-6-year-old children can solve for an unknown addend in a non-symbolic arithmetic problem (Kibbe and Feigenson, 2015, 2017), this previous work tested children using a single trial in which children were asked to identify which of two quantities (a target quantity, or a distractor quantity that differed from the target quantity by at least a 1:2 ratio) matched the quantity of the unknown addend. There has been no work examining the precision of non-symbolic unknown-addend operations in children. An additional goal of the present study was therefore to shed light on the operational precision of unknown-addend operations between ages 4 and 6.

We had several hypotheses. First, since previous work showed increased representational precision between the ages of 4 and 6 (e.g., Halberda and Feigenson, 2008), we expected to observe increased representational (Baseline condition) and operational (Addition and Unknown-addend conditions) precision with age. With respect to how age might interact with different operations, we outlined several possibilities. If a primary source of noise in ANS operations comes from the precision of the inputs to these operations, then we would expect the development of ANS operational precision (within both Addition and Unknown-Addend conditions) to follow the same trajectory as the development of ANS representational precision (as measured in the Baseline condition). Such a pattern would suggest that children’s ANS operational abilities are consistent over time, while the representational precision of the ANS undergoes developmental change. On the other hand, if children’s adeptness with non-symbolic operations increases differentially over time, we may expect to observe different developmental trajectories for different non-symbolic operations as compared to the Baseline condition.

## 2. Method

### 2.1. Participants

Seventy-two 4- to 6-year-old U.S. children completed all study procedures. The sample included 24 4-year-olds (mean age: 4 years 6 months, range: 4 years 0 months– 4 years 11 months, 15 girls), 24 5-year-olds (mean age: 5 years 6 months, range: 5 years 0 months – 5 years 11 months, 11 girls), and 24 6-year-olds (mean age: 6 years 8 months, range: 6 years 0 months – 7 years 0 months, 15 girls). The sample size was determined prior to data collection based on a power analysis using G*Power 3.1 for a repeated measures ANOVA with Condition as a within-participants factor and Age Group as a between-participants factor (*α* = 0.05, 1-β = 0.9, effect size *f* = 0.2, suggested total sample *n* = 69). Ten children (two 4-year-olds, three 5-year-olds, five 6-year-olds) were tested in the lab pre-COVID. The remaining 62 children were tested remotely via Zoom videoconferencing software due to the ongoing COVID-19 pandemic. An additional six children (one 4-year-old, four 5-year-olds, one 6-year-old) were tested but excluded from analysis because they declined to complete the study.

Forty-two out of the 72 caregivers completed an optional demographics form. Children were reported by their parents as Asian (5), Asian/White (4), Black (1), Middle Eastern/Arab (1), or White (31). One of the children was reported as being Hispanic/Latinx. 41/42 children came from households with at least 1 parent who had a college degree or higher. Children were recruited from the greater Boston area via mailing lists and local child-friendly events, and all families received a $10 Amazon gift card for their participation. All study procedures were approved by the Boston University Charles River Campus Institutional Review Board.

### 2.2. Stimuli

Stimuli consisted of animations presented in Keynote presentation software. In each condition, children viewed arrays of green or orange dots (“buttons”) presented on the left and right sides of the screen, respectively. Dots varied in size (on a 13.3-inch screen, the diameters of the dots were 0.1 cm, 0.2 cm, 0.3 cm, 0.4 cm, 0.5 cm, and 0.6 cm). The proportion of area that a dot took up the screen ranged from 0.0015 to 0.0557%. The dots were distributed pseudo-randomly in space (to minimize grouping or crowding) and did not overlap. The contour area of the arrays of dots within each trial was approximately equated to discourage participants from relying on the overall contour area as a quantity cue. Dot arrays could be occluded by an animated blue “cup.” To motivate children and help them to understand the task, we used a cartoon alligator and a cartoon cheetah and told children that these characters were having a contest to see who had the most buttons.

Children who participated in the lab (*n =* 10) viewed the stimuli on a MacBook Air 13-inch screen in a quiet laboratory testing room. Children who participated online (*n =* 62) viewed the stimuli via the screen-sharing function in Zoom on a home device (58 families used a laptop or a desktop computer, 4 used a tablet with a screen at least 10 inches). Full stimuli are available at https://osf.io/vxma3/.

### 2.3. Design

The experiment consisted of three conditions: a Baseline condition, an Addition condition, and an Unknown-addend condition, presented in blocks. In trials in the Baseline condition, children observed an array which was then occluded, and were asked to compare their representation of the occluded array to a visible comparison array. In trials in the Addition condition, children observed an array which was then occluded, and then observed another array which then moved behind the same occluder. Children were asked to compare their representation of the *sum* of the two arrays to a visible array. In trials in the Unknown-addend condition, children observed an array which was then occluded, and then revealed to have been incremented by an unobserved quantity. Children had to compare their representation of the *difference* between the final quantity and the initial quantity (that is, they had to compute the quantity that had been added) to a visible array.

We manipulated the ratio between the transformed array and the visible comparison array (comparison ratios: 0.5, 0.67, 0.75, 0.80). These ratios were chosen to be similar to comparison ratios used in previous work examining 5-year-olds’ accuracy in non-symbolic addition problems (Barth et al., 2006). Whether the quantity to be compared (the occluded array in Baseline trials, the summed total in Addition trials, the unknown addend in the Unknown-addend trials) was larger or smaller than the comparison array was counterbalanced across trials.

Children completed 14 test trials per condition (two trials per ratio 0.5, four trials per ratios 0.67, 0.75, and 0.8). All children completed the Baseline condition first, followed by the Addition and Unknown-addend conditions, with order of these two conditions counterbalanced across participants. Children completed one of two pseudorandomized test trial orders (see Supplementary Tables S1–S3 for details).

### 2.4. Procedure

The procedures for children tested in the lab and online were identical except that for children who participated online, caregivers first completed an online-set-up procedure before children began the study. The experimenter instructed caregivers to enter full-screen mode, hide the self-view panel, and place the experimenter’s video panel in the top center of the screen. The online set-up therefore provided a virtual face-to-face interactive environment.

#### 2.4.1. Baseline condition

##### 2.4.1.1. Practice

The experimenter first introduced children to two characters, Gator and Cheetah. The experimenter told children that Gator and Cheetah “are having a button contest. To see who is winning the contest, you need to help me figure out who has more buttons.” To ensure that children were paying attention, the experimenter drew children’s attention to each character by playing a jumping animation and asked children to name each character.

Next, the experimenter presented Gator, Gator’s cup, and a set of green buttons on the left side of the screen and said “These are Gator’s buttons. Gator likes to hide his buttons inside his cup. Like this.” Gator’s cup then moved to cover the pile so that the buttons were completely occluded. The experimenter then presented Cheetah and a set of orange buttons on the right side of the screen, and said “This is Cheetah, and his buttons.” The experimenter then asked, “Can you tell me who has more buttons, Gator or Cheetah?” After children responded, the experimenter showed the actual amount in Gator’s cup. If the child answered correctly, the experimenter said, “Good job!.” If the child answered incorrectly, the experimenter said, “Actually Gator/Cheetah (the correct answer) has more buttons. That’s ok. Let us try another one!.” The speed of the practice trial was controlled by the experimenter who made sure that children understood each step before proceeding to the next.

Children completed two practice trials. In both practice trials, the ratio between Gator’s and Cheetah’s arrays was 0.5; Gator’s array was larger in one trial, while Cheetah’s was larger in the other trial. The average proportion correct in the practice trials was 92%. Regardless of children’s performance in these trials, the experimenter proceeded to the Test trials.

##### 2.4.1.2. Test

**Figure 1:**
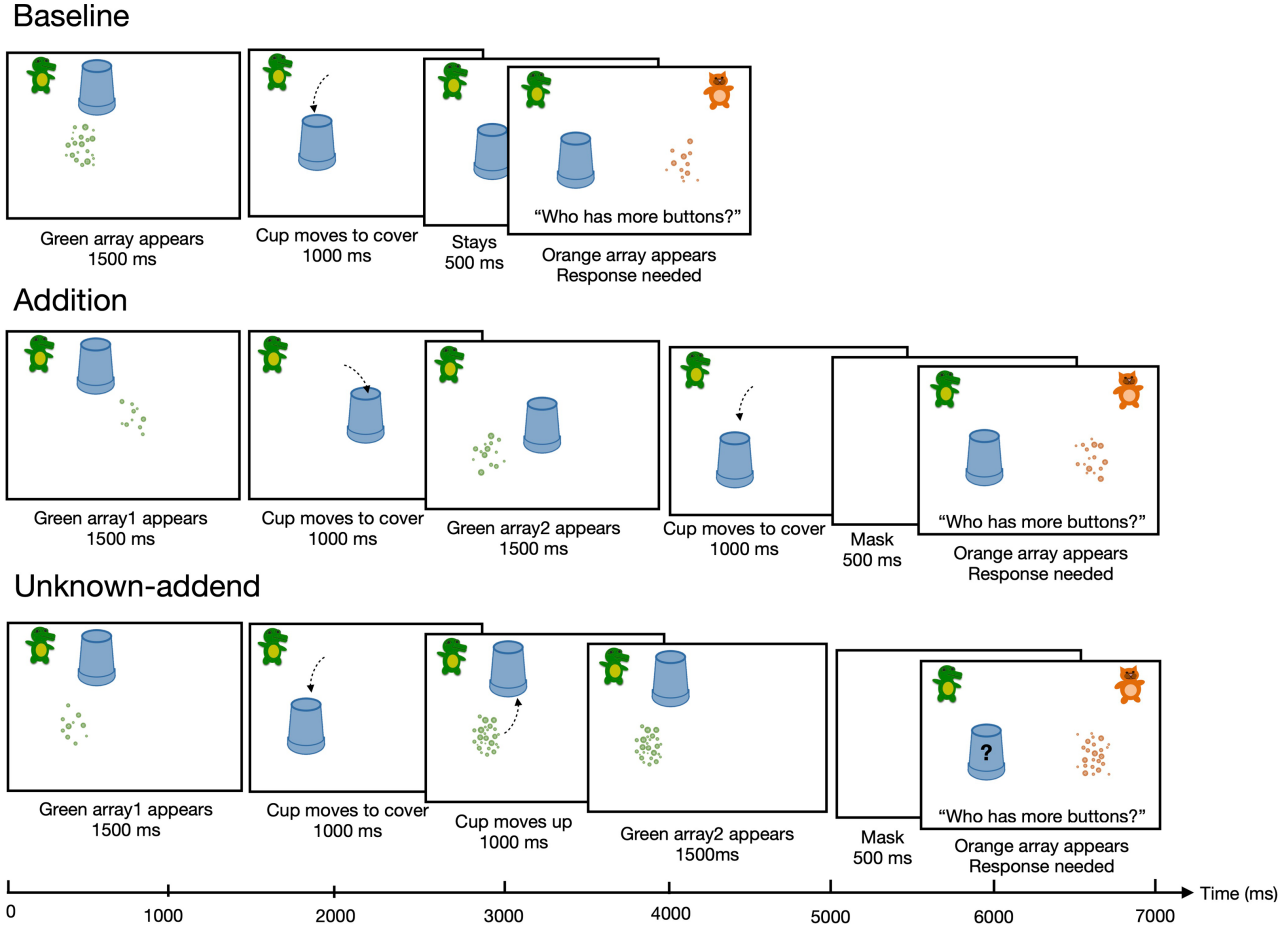
The procedure of the Baseline, Addition, and Unknown-addend conditions were depicted in the top, middle, and bottom rows. Children were asked the same questions at the response phase. Each array of to-be-manipulated dots was presented for 1,500 ms in all three conditions. A blank mask was presented before the response phase to disrupt the maintenance of the numerical representation at the approximate same location during the computation phase in Addition and Unknown-addend conditions.

Test trials proceeded similarly to the practice trials, except that Gator’s (green) buttons were presented for a fixed duration of 1,500 ms before they were occluded by the cup. The occlusion animation took 1,000 ms from the start of the movement of the cup to the complete occlusion of the array. Gator’s array then remained occluded for 500 ms, after which Cheetah’s comparison array appeared and remained visible until children gave their response. The experimenter then advanced to the next trial. Whether Gator’s or Cheetah’s array was larger was counterbalanced across trials. Children received no feedback on Test trials. Figure 1, top panel, shows a timeline of an example Baseline test trial.

#### 2.4.2. Addition condition

#### 2.4.3. Practice

The experimenter said, “Let us try something different. We still have to decide who has more buttons, Gator or Cheetah. But this time, Gator is going to add *two* piles of buttons in his cup instead of just one. Like this^1^.” The experimenter then stepped through the animations. Children observed a set of green buttons appear on the left side of the screen. Gator’s cup then moved to cover the array. A second set of green buttons then appeared, and Gator’s cup moved to occlude those buttons as well, taking with it the first array. The complete animated sequence thus gave the impression that the cup “scooped up” the two sets of buttons sequentially. After the cup moved to its final location, children observed a blank mask screen. The blank mask was added to keep it consistent with the procedures in the Unknown-addend condition. The response screen then appeared, with Gator and his cup presented on the left side of the screen and Cheetah and his array of buttons on the right side of the screen. Children were asked which character had more buttons. Thus, children had to compare the summed total of Gator’s two sets of buttons to Cheetah’s visible set of buttons.

In both practice trials, children received feedback on their responses: after children gave their response, the experimenter revealed the total quantity in Gator’s cup and told children whether they were correct. The ratio between the sum of Gator’s two arrays and Cheetah’s array was 0.5 in both trials. Gator’s total was larger than Cheetah’s array in one trial, and Cheetah’s array was larger than Gator’s total in the other trial. Children’s average proportion correct in the practice trials was 86%. All animations in the practice trials were controlled by the experimenter.

*Test.* Test trials proceeded similarly to the practice trials, except that the animation sequence was presented with fixed timings rather than being paced by the experimenter. Children observed the first array of green buttons appear on the left side of the screen. After 1,500 ms, Gator’s cup moved to cover the array (1,000 ms total motion). The second array of green buttons then appeared and remained visible for 1,500 ms, after which Gator’s cup moved to occlude the second array (1,000 ms total motion), taking the first array with it. After Gator’s cup moved to its final location, children observed a blank mask screen for 500 ms. Children were then presented with Gator’s cup and Cheetah’s visible array, and were asked to choose which character had more buttons. Children received no feedback on the test trials. Figure 1, middle panel, shows a timeline of an example Addition test trial.

#### 2.4.4. Unknown-addend condition

##### 2.4.4.1. Practice

The experimenter first introduced the Unknown-addend condition by saying “Let us try something different. We still have to try to figure out who has more buttons, Gator or Cheetah. But this time it’s going to get a little tricky.”

The Unknown-addend condition then began with two familiarization trials designed to introduce children to the fact that Gator’s cup already contains buttons at the start of each trial. The experimenter showed children Gator and his cup, and told children “Now, Gator has already got some buttons in his cup.” The experimenter then advanced the animation, and the cup became transparent, revealing the buttons “inside” the cup. The experimenter told children, “See? Gator has already got some buttons in his cup” She then advanced the animation and the cup returned to opacity. She then showed children a visible array of green buttons on the left side of the screen, and said “Gator is going to be sneaky. Here’s a pile of buttons. Those buttons do not belong to Gator. But he is going to mix his buttons up with those buttons that do not belong to him. Like this.” The experimenter advanced the animation so that Gator’s cup occluded the array of green buttons and then returned to its original location, leaving behind the buttons that had been inside the cup. The experimenter then said “See? Now they are all mixed together. But we got to see what was in his cup, so we know how many buttons he had. To see if Gator is winning the button contest, we only want to count the buttons that were in Gator’s cup in the first place, since those are Gator’s buttons. We do not want to count the buttons that do not belong to him.” Before presenting the response screen where Gator’s cup was displayed with Cheetah’s quantities side-by-side, a blank mask screen was displayed to prevent children from continuously representing the visible buttons in the second set and using this final quantity in Gator’s demonstration to compare with Cheetah’s quantity. In the response screen, the experimenter showed Cheetah and his buttons on the right side and asked, “Can you tell me who has more buttons, Gator or Cheetah?” Children then completed a second familiarization trial in which the quantity in Gator’s cup was revealed before the contents were added to the set. On both trials, the ratio between the quantity in Gator’s cup and Cheetah’s set was 0.5. After children responded, the experimenter advanced the animation to reveal the buttons inside Gator’s cup and gave children feedback on their responses. The average proportion correct in the familiarization trials was 86%.

Children then completed two practice trials, which were similar to the familiarization trials except that the quantity inside Gator’s cup was not made visible to children. Instead, children had to estimate the quantity that had been inside Gator’s cup by comparing the quantity before the cup was added to the quantity after the cup was added. The speed of the presentation was controlled by the experimenter. The average proportion correct of children’s responses in the first attempt was 64%. Given the complexity of the instructions compared to the other conditions, if children responded incorrectly, the experimenter replayed the trial, stepping through the animations again to ensure that children understood the task. All children responded correctly after seeing a trial the second time. All animations in familiarization and practice trials were controlled by the experimenter.

##### 2.4.4.2. Test

The test trials followed the same procedures as the practice trials except the timing of each animation was fixed. The first set of green buttons was presented for 1,500 ms, the cup then moved to cover the first set for 1,000 ms. After a delay of 1,000 ms, the cup moved back to its original position next to Gator (movement duration 1,000 ms). The second set of green buttons were presented for 1,500 ms. Children then viewed a blank screen mask for 500 ms, followed by an image of Gator’s cup and Cheetah’s visible array, and were asked to choose who had more buttons. The experimenter did not provide feedback after the child gave an answer in each trial. Figure 1, bottom panel, shows a timeline of an example Unknown-addend test trial.

## 3. Results

**Figure 2:**
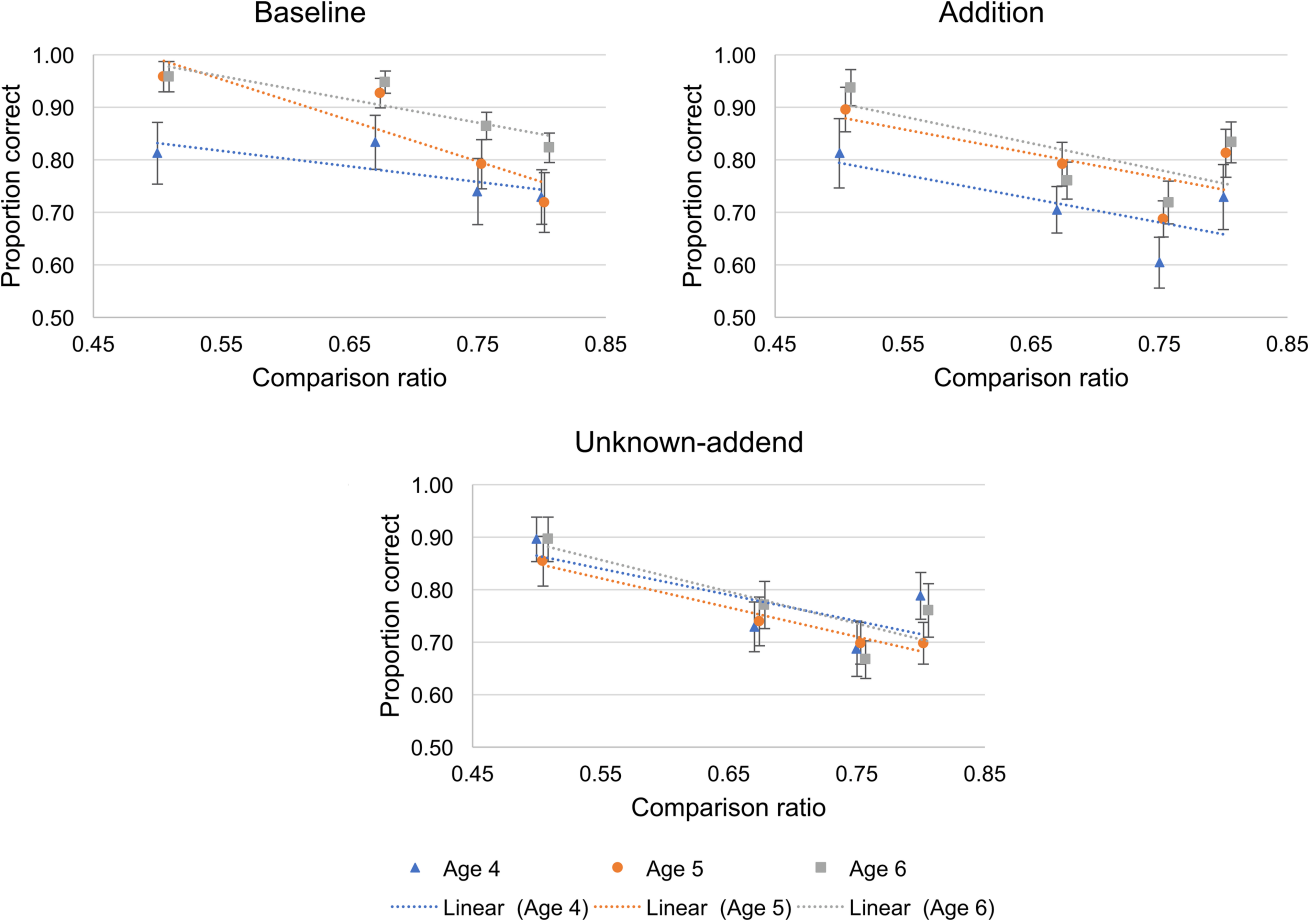
Children’s mean proportion correct as a function of comparison ratio (0.50, 0.67, 0.75, 0.80) in the three conditions (Baseline, Addition, Unknown-addend). The blue triangles show performance in 4-year-old children, the orange dots show performance in 5-year-old children, and the grey squares show performance in 6-year-old children. Error bars indicate ± 1 standard error of the mean. Dotted lines show the best fitting lines for each age group.

We coded children’s response on each trial as 1 if they were correct and 0 if they were incorrect. We then computed children’s mean proportion correct for each ratio within each condition in each age group.^2^ Analyses were conducted on these means (see Figure 2; Supplementary Table S4 for means for each ratio and condition, broken down by age group). All analyses were conducted using SPSS. Data on which the analyses were conducted can be found at https://osf.io/vxma3/.

We first examined whether children’s accuracy across comparison ratios and conditions differed between Testing sites (lab: *n* = 10; online: *n* = 62). We performed a repeated measures ANOVA with Testing sites as between-participants factor and Condition (Baseline, Addition, Unknown-addend) and comparison Ratio (0.50, 0.67, 0.75, 0.80) as within-participants factors (we did not include Age Group as a between-participants factor due to the small number of children included in the lab). Results showed no significant effects of Testing site [*F*(1,70) = 2.96, *p* = 0.090, η_p_^2^ = 0.041] and no interaction effects [all *F*(1,70) <1.87, *p* > 0.176, η_p_^2^ < 0.026]. We therefore did not include testing site in further analyses. To examine whether children’s accuracy across comparison ratios and conditions varied as a function of counterbalancing assignment, we ran a repeated measures ANOVA with Condition Order (Baseline – Addition – Unknown-Addend or Baseline – Unknown-Addend – Addition), Trial Order (pseudo-randomized trial order: Order 1 or Order 2), and Age Group (4-, 5-, 6-year-olds) as between-participants factors, and Condition and Ratio as within-participants factors. Results revealed no significant effects of Condition Order [*F*(1,60) = 1.60, *p* = 0.211, η_p_^2^ = 0.026], Trial Order [*F*(1,60) = 0.185, *p* = 0.668, η_p_^2^ = 0.003], or interaction effect between these variables [all *F* < 0.552, *p* > 0.460, η_p_^2^ < 0.009] on children’s performance. We therefore dropped these factors in the remaining analyses.

To examine the effect of condition, comparison ratios, and age on children’s accuracy, we conducted a repeated measures ANOVA on children’s mean proportion correct at each comparison ratio with Ratio (0.50, 0.67, 0.75, 0.80) and Condition (Baseline, Addition, Unknown-addend) as within-participants factors and Age Group (4-, 5-, 6-year-olds) as a between-participants factor. We observed a main effect of Ratio [*F*(3, 207) = 29.47, *p* < 0.001, η_p_^2^ = 0.299]; children’s mean proportion correct decreased as the ratio between the transformed array and the comparison array approached 1:1, confirming that children were using magnitude representations, rather than precise quantity representations (obtained by, e.g., counting) to solve these tasks.^3^ We also observed a main effect of Condition [*F*(2, 138) = 8.62, *p* < 0.001, η_p_^2^ = 0.111] subsumed under an interaction between Condition and Ratio [*F*(6, 414) = 4.04, *p* < 0.001, η_p_^2^ = 0.055]. We also observed a main effect of Age Group [*F*(2, 69) = 3.73, *p* = 0.029, η_p_^2^ = 0.097]; (no other interactions were statistically significant; *ps* > 0.066).

We followed up this analysis with planned separate repeated measures ANOVAs on Baseline, Addition, and Unknown Addend conditions, with Ratio (0.50, 0.67, 0.75, 0.80) as a within-participants factor and Age Group as a between-participants factor. In the Baseline condition, we observed a main effect of Ratio [*F*(3, 207) = 12.16, *p <* 0.001, η_p_^2^ = 0.15], a main effect of Age Group [*F*(1, 69) = 4.5, *p =* 0.015, η_p_^2^ = 0.115], and no Ratio by Age Group interaction [*F*(6, 207) = 0.828, *p =* 0.55, η_p_^2^ = 0.023]. We observed a similar pattern of results in the Addition condition, with a main effect of Ratio [*F*(3, 207) = 12.93, *p <* 0.001, η_p_^2^ = 0.158], a main effect of Age Group [*F*(1, 69) = 3.96, *p =* 0.024, η_p_^2^ = 0.103], and no Ratio X Age Group interaction [*F*(6, 207) = 0.19, *p =* 0.981, η_p_^2^ = 0.005]. However, in the Unknown-addend condition, we observed only the main effect of Ratio [*F*(3, 207) = 12.61, *p <* 0.001, η_p_^2^ = 0.154]; there was no main effect of Age Group [*F*(1, 69) = 0.32, *p =* 0.73, η_p_^2^ = 0.009] and no Ratio X Age Group interaction [*F*(6, 207) = 0.50, *p =* 0.809, η_p_^2^ = 0.014]. These results suggest that children’s performance in Baseline and Addition conditions increased with age, while children’s performance in the Unknown-Addend condition did not improve with age. These results are illustrated in Figure 2.

Next, we investigated whether individual children’s performance in one condition correlated with their performance in the other conditions. We computed correlations between children’s performance at each ratio between conditions, controlling for participant. We observed significant correlations between the Baseline and Addition conditions (*r* = 0.159, *p* = 0.007), Baseline and Unknown Addend conditions (*r* = 0.165, *p* = 0.005), and between Addition and Unknown Addend conditions (*r* = 0.127, *p* = 0.031).

Finally, we compared children’s mean proportion correct for each comparison ratio in each condition to chance (0.5) using one-sample *t* tests. To correct for multiple comparisons within each age group and condition, we set the alpha criterion for statistical significance to 0.017. Children’s mean proportion correct performance was above chance for each ratio in each condition [all *t*(23) > 2.87, *p* < 0.002, *d* > 1.48, BF_10_ > 21], except for 4-year-old’s performance in ratio 0.75 of the Addition condition [*t*(23) = 2.15, *p* = 0.042, *d* = 0.90, BF_10_ = 1.2], which did not meet our strict criterion for statistical significance (see Supplementary Table S4 for the results of all comparisons).

We next asked whether children may have engaged in response strategies that did not require them to actually perform specific operations over ANS representations. That is, we asked whether children could have achieved above-chance performance without actually performing a summation operation in the Addition condition, or without performing a difference operation in the Unknown-addend condition. To do so, we performed a series of targeted analyses, detailed in the Supplementary material, to explore whether children were engaging in such response strategies (see Barth et al., 2006, for a similar approach). Results of these analyses suggest that children were indeed performing summation and unknown-addend operations in our task, and were not likely using alternative, non-computational strategies.

## 4. Discussion

We examined the development of the precision of 4- to 6-year-old U.S. children’s solutions to two different non-symbolic arithmetic operations. Children completed three non-symbolic numerical comparison tasks in which they were asked to judge which of two arrays was larger. In the Baseline condition, children compared their representation of an occluded array to a comparison array. In the Addition condition, children were asked to compute the sum of two sequentially occluded arrays and compare the sum to a comparison array. In the Unknown-addend condition, children were asked to infer the quantity of an unknown addend by computing the difference between the initial array (before occlusion) and the final array (after the unknown quantity was added), and compare this quantity to the comparison array. This allowed us to examine the precision of solutions to different kinds of approximate arithmetic operations within participants, during a developmental period in which the precision of the ANS is undergoing substantial development (Halberda and Feigenson, 2008).

We found that young children could solve non-symbolic comparison, addition, and unknown-added problems with fairly high fidelity, as suggested by their above-chance performance at a range of ratios. Further trial-by-trial analysis confirmed that children were unlikely to be using non-operational strategies in the Addition and Unknown-addend conditions. These results are consistent with previous work showing that children as young as 4 years can perform non-symbolic addition and unknown-addend operations (e.g., Barth et al., 2006; Gilmore et al., 2010; Kibbe and Feigenson, 2015, 2017; Cheng and Kibbe, 2023).

We observed substantial development of the representational precision of the ANS across our age range in our Baseline condition, in which children were asked to compare a single array stored in working memory to a visible comparison array, consistent with previous work that asked children to compare two visible arrays (e.g., Halberda and Feigenson, 2008). We also observed developmental change in the precision of the outputs of addition operations; with age, children’s operational precision for summing two, sequentially presented arrays increased. However, we did not observe developmental improvements in the precision of unknown-addend operations across our age range. Children’s performance in the Addition and Unknown-addend conditions separately was correlated with their performance in the Baseline condition, and their performance in Addition and Unknown-addend conditions also was correlated (although correlations among conditions were relatively weak).

Our findings provide new insights into the development of ANS operational precision between the ages of 4 and 6 years. Together, these results suggest different trajectories for the development of the precision of different operations over ANS representations. The relatively weak correlation between children’s performance in the comparison condition and the ANS operation conditions suggested that the development of the *representational* precision of the ANS may be *one,* but not the *only* source of developmental change in children’s ability to perform non-symbolic operations over ANS representations, at least in the conditions we tested here. While identifying the factors that contribute to these developmental changes is beyond the scope of the current study, here we propose several potential sources of development of ANS operational precision.

First, children’s ability to perform the operations themselves could be developing during this period. While both the addition and unknown-addend problems in our task required children to hold ANS representations in mind and perform an operation over those representations, the process for solving for an unknown addend is conceptually different from performing an addition operation. Addition can be accomplished by maintaining a running total of items as they are encountered, while solving for an unknown addend requires that children (a) perform a subtraction operation (which previous work has shown results in less precise outputs than addition operations in 5-year-olds; Barth et al., 2016) and (b) to start with the result and infer the solution backward (which can be extremely challenging for children, even into the school years; e.g., Booth, 1988; Riley and Greeno, 1988; Filloy and Rojano, 1989; Kieran, 1992; Koedinger et al., 2008). Our results suggest the possibility that the ability to “back-solve” over ANS representations undergoes more protracted development than the ability to maintain a running total. Further work is needed to tease apart potential computational sources of developmental change by comparing more operations in a within-subjects design (including subtraction).

Second, general cognitive functions are undergoing development between 4 and 6 years (Espy et al., 1999; Happaney et al., 2004; Cowan et al., 2005; Fuhs and McNeil, 2013; Gilmore et al., 2013; Heyes et al., 2016; Simmering and Miller, 2016; Guillory et al., 2018). In particular, the capacity both to store and to *manipulate* information in working memory increases substantially during this period (Riggs et al., 2006; Simmering, 2012; Pailian et al., 2016; Cheng and Kibbe, 2022). While both the Addition and Unknown-addend conditions required children to store ANS representations in working memory and perform operations over ANS representations, the tasks made slightly different demands on working memory due to the different ways in which the operations can be accomplished (see Cheng and Kibbe, 2023). In the Addition condition, children could store the first set in working memory until they observed the second set, at which point they could increment their representation by the quantity added, discarding any memory of the first ANS representation completely. They then could store the updated ANS representation in working memory to compare to the visible comparison array. By contrast, in the Unknown-addend condition, children had to hold a representation of the initial array in working memory until they observed the final quantity, and then perform an operation over the ANS representation in working memory and the ANS representation of the final quantity to derive the difference between the two. Thus, children had to perform a more complex manipulation of the stored ANS representations in the Unknown-addend condition compared to the Addition condition. It is possible that each step of the computational process for each operation, including storing and manipulating ANS representations, may impose different levels of cognitive demands, impacting the precision of the outputs. Furthermore, storing an additional array of quantities and executing more steps in non-symbolic operational manipulations (Addition and Unknown-addend conditions) compared to storing a single array with non-operational manipulations (Baseline condition) may introduce more chances for processing errors to occur. It is also possible that, with the development of working memory, ANS representations may be stored with higher fidelity and longer term, and may be manipulated without excessive degradation. Further work is needed to examine how working memory development contributes to the development of ANS operational precision.

When young children are learning symbolic math, they succeed at symbolic addition problems (e.g., 12 + 7 = x) much earlier than symbolic unknown addend problems (e.g., 12 + x = 19), which can take years to master and can cause difficulties even into the college years (e.g., Booth, 1988; Riley and Greeno, 1988; Filloy and Rojano, 1989; Kieran, 1992; Koedinger et al., 2008). Our results suggest this developmental lag is present in non-symbolic operations as well, providing a potential foothold for future work to examine whether domain-general cognitive sources impact children’s unknown-addend reasoning. By providing basic data on the developmental time course of different non-symbolic operations, the present study could inform future work that could explore the role of using non-symbolic arithmetic-like problems to support the acquisition of different types of symbolic operations.

Our study also has some limitations. Firstly, we observed an uptick performance in ratio 0.80 compared to ratio 0.75 in Addition and Unknown-addend conditions, as captured in the interaction effect between Condition and Ratio. This may be due to an issue with the way the trials were constructed: the numerosities used in one of the trials were much larger than in the other trials, which may have artificially deflated children’s performance on that ratio. Previous work suggested that children’s representational precision is negatively related to the magnitude of the numerosity (Gallistel and Gelman, 1992; Whalen et al., 1999). We suspect that children’s lower performance in this single trial in ratio 0.75 is likely due to the noisier representations that children had to manipulate to operate over the relatively larger quantities on that trial. In an exploratory analysis, we removed that trial completely and repeated all analyses reported in the Results section. Doing so did not meaningfully change any of the outcomes of these analyses. Further work is needed to examine the contribution of numerosity magnitudes on children’s operational precision.

Secondly, while we varied the size of the individual items in each stimulus set to promote children’s focus on quantity over non-numerical perceptual features like cumulative surface area or density of the arrays, we did not precisely control for such features. These continuous quantity variables have been found to affect numerical estimation in non-symbolic tasks (Gebuis and Reynvoet, 2012; Leibovich et al., 2017). Future work would implement more strict control of the non-numerical visual cues to study non-symbolic arithmetic operations (see De Marco and Cutini, 2020). We also tested only two (strategically chosen) non-symbolic arithmetic operations in order to minimize participant fatigue in our within-participants design. Future work would explore a range of non-symbolic arithmetic operations, including contrasting subtraction and unknown-addend operations, to gain further insights into the interactions between ANS representational and operational precision.

In our study, we have embedded the tasks within a social context: to engage young participants, we introduced the tasks as a “button competition” between two animal characters Gator and Cheetah and encouraged children to find out who has more buttons. In the Unknown-addend condition, we described Gator as a “sneaky” character who wanted to pretend to have more. While examining the role of social context is beyond the scope of the current study, previous work has shown that young children’s numerical computation ability is impacted by social scenarios such as sharing (Hamamouche et al., 2020). Future work can investigate the impact of the types of social contexts in which tasks are embedded and/or the characters’ social properties on children’s numerical computation and reasoning.

In sum, we found evidence for differential development of the precision of different operations over numerical representations of large sets of items– specifically, addition and unknown-addend operations – between the ages of 4 and 6 years in a sample of US children. Our results suggest that both representational and operational precision must be considered to gain a complete understanding of the large set quantification in development.

## Data availability statement

The datasets presented in this study can be found in online repositories. The names of the repository/repositories and accession number(s) can be found in the article/Supplementary material.

## Ethics statement

The studies involving humans were approved by Boston University Institutional review board. The studies were conducted in accordance with the local legislation and institutional requirements. Written informed consent for participation in this study was provided by the participants’ legal guardians/next of kin.

## Author contributions

CC: Conceptualization, Formal analysis, Project administration, Visualization, Writing – original draft, Writing – review & editing. MMK: Conceptualization, Formal analysis, Funding acquisition, Supervision, Writing – review & editing.

## Footnotes

^1^Note that when the Addition followed the Unknown-addend condition, the experimenter highlighted the difference between the conditions by emphasizing that “now *both* piles of green buttons are Gator’s.”

^2^Two children declined to complete all trials: one participant completed 3 out of 4 trials for ratio 0.67 and ratio 0.75 in the Addition condition; the other participant completed 3 out of 4 trials for ratio 0.75 in the Addition condition and 3 out of 4 trials for ratio 0.8 in the Unknown-addend condition.

^3^We noticed there was a slight uptick in performance between ratio 0.75 and ratio 0.80. Inspecting children’s performance on each trial, we found that children performed significantly worse in one trial [Addition (14 + 22) vs. 27, *M* = 48%] than the rest of the trials (65% ~ 88%) in ratio 0.75. This trial required children to operate over larger quantities relative to the other trials at 0.75 and 0.8 (see Supplementary Table S4). See Discussion section for further discussion.
