# Supplementary material for: Development of precision of non-symbolic arithmetic operations in 4-6-year-old children
Source: Front Psychol. 2023 Nov 15;14:1286195. doi: 10.3389/fpsyg.2023.1286195 (PMC10684939; doi:10.3389/fpsyg.2023.1286195)
Supplement: Supplementary file 1 [file Data_Sheet_1.docx]

Supplementary Material

The datasets analyzed for this study can be found in the OSF repository (<https://osf.io/vxma3>).

# Quantity sheet

## Baseline condition

|  | Order 1 | | | Order 2 | | |
| --- | --- | --- | --- | --- | --- | --- |
| Trial | Ratio | Gator | Cheetah | Ratio | Gator | Cheetah |
| 1 | .50 | 10 | 20 | .50 | 18 | 9 |
| 2 | .67 | 21 | 14 | .67 | 12 | 18 |
| 3 | .67 | 24 | 16 | .67 | 16 | 24 |
| 4 | .67 | 16 | 24 | .67 | 24 | 16 |
| 5 | .80 | 15 | 12 | .80 | 20 | 25 |
| 6 | .75 | 12 | 16 | .75 | 20 | 15 |
| 7 | .80 | 10 | 8 | .80 | 28 | 35 |
| 8 | .80 | 20 | 25 | .80 | 15 | 12 |
| 9 | .75 | 20 | 15 | .75 | 12 | 16 |
| 10 | .50 | 18 | 9 | .50 | 10 | 20 |
| 11 | .75 | 36 | 27 | .75 | 27 | 36 |
| 12 | .75 | 27 | 36 | .75 | 36 | 27 |
| 13 | .80 | 28 | 35 | .80 | 10 | 8 |
| 14 | .67 | 12 | 18 | .67 | 21 | 14 |

Table S1. Quantities used (and ratios between them) in Trial Orders 1 and 2 in the Baseline condition.

## Addition condition

|  | Order 1 | | | | Order 2 | | | |
| --- | --- | --- | --- | --- | --- | --- | --- | --- |
| Trial | Ratio | Gator Set1 | Gator Set2 | Cheetah | Ratio | Gator Set1 | Gator Set2 | Cheetah |
| 1 | .50 | 6 | 12 | 9 | .50 | 4 | 6 | 20 |
| 2 | .67 | 7 | 5 | 18 | .67 | 11 | 10 | 14 |
| 3 | .67 | 10 | 6 | 24 | .67 | 10 | 14 | 16 |
| 4 | .67 | 10 | 14 | 16 | .67 | 10 | 6 | 24 |
| 5 | .80 | 13 | 7 | 25 | .80 | 7 | 8 | 12 |
| 6 | .75 | 8 | 12 | 15 | .75 | 5 | 7 | 16 |
| 7 | .80 | 13 | 15 | 35 | .80 | 6 | 4 | 8 |
| 8 | .80 | 7 | 8 | 12 | .80 | 13 | 7 | 25 |
| 9 | .75 | 5 | 7 | 16 | .75 | 8 | 12 | 15 |
| 10 | .50 | 4 | 6 | 20 | .50 | 6 | 12 | 9 |
| 11 | .75 | 12 | 15 | 36 | .75 | 14 | 22 | 27 |
| 12 | .75 | 14 | 22 | 27 | .75 | 12 | 15 | 36 |
| 13 | .80 | 6 | 4 | 8 | .80 | 13 | 15 | 35 |
| 14 | .67 | 11 | 10 | 14 | .67 | 7 | 5 | 18 |

Table S2. Quantities used (and ratios between them) in Trial Orders 1 and 2 in the Addition condition.

## Unknown-addend condition

|  | Order 1 | | | | | Order 2 | | | | |
| --- | --- | --- | --- | --- | --- | --- | --- | --- | --- | --- |
| Trial | Ratio | Starting Set | Ending Set | Gator | Cheetah | Ratio | Starting Set | Ending Set | Gator | Cheetah |
| 1 | .50 | 4 | 14 | 10 | 20 | .50 | 6 | 24 | 18 | 9 |
| 2 | .67 | 11 | 32 | 21 | 14 | .67 | 7 | 19 | 12 | 18 |
| 3 | .67 | 10 | 34 | 24 | 16 | .67 | 10 | 26 | 16 | 24 |
| 4 | .67 | 10 | 26 | 16 | 24 | .67 | 10 | 34 | 24 | 16 |
| 5 | .80 | 7 | 22 | 15 | 12 | .80 | 13 | 33 | 20 | 25 |
| 6 | .75 | 5 | 17 | 12 | 16 | .75 | 8 | 28 | 20 | 15 |
| 7 | .80 | 6 | 16 | 10 | 8 | .80 | 13 | 41 | 28 | 35 |
| 8 | .80 | 13 | 33 | 20 | 25 | .80 | 7 | 22 | 15 | 12 |
| 9 | .75 | 8 | 28 | 20 | 15 | .75 | 5 | 17 | 12 | 16 |
| 10 | .50 | 6 | 24 | 18 | 9 | .50 | 4 | 14 | 10 | 20 |
| 11 | .75 | 14 | 50 | 36 | 27 | .75 | 12 | 39 | 27 | 36 |
| 12 | .75 | 12 | 39 | 27 | 36 | .75 | 14 | 50 | 36 | 27 |
| 13 | .80 | 13 | 41 | 28 | 35 | .80 | 6 | 16 | 10 | 8 |
| 14 | .67 | 7 | 19 | 12 | 18 | .67 | 11 | 32 | 21 | 14 |

Table S3. Quantities used (and ratios between them) in Trial Orders 1 and 2 in the Unknown-addend condition.

# One-Sample t tests results

| **Age** | **Condition** | **Ratio** | ***M*** | ***SD*** | ***t*(23)** | ***p*** | ***d*** | **BF_10_** |
| --- | --- | --- | --- | --- | --- | --- | --- | --- |
| **4** | Baseline | .50 | .81 | .29 | 5.32 | <.001 | 2.22 | >1000 |
|  |  | .67 | .79 | .30 | 6.49 | <.001 | 2.71 | >1000 |
|  |  | .75 | .70 | .34 | 3.81 | <.001 | 1.59 | 35.71 |
|  |  | .80 | .73 | .25 | 4.41 | <.001 | 1.84 | 143 |
|  | Addition | .50 | .81 | .32 | 4.73 | <.001 | 1.97 | 333 |
|  |  | .67 | .72 | .23 | 4.63 | <.001 | 1.93 | 250 |
|  |  | .75 | .60 | .24 | 2.15 | .042 | .90 | 1.2 |
|  |  | .80 | .73 | .30 | 3.70 | .001 | 1.54 | 29 |
| Unknown-addend | | .50 | .90 | .21 | 9.35 | <.001 | 3.90 | >1000 |
|  |  | .67 | .73 | .23 | 4.84 | <.001 | 2.02 | 333 |
|  |  | .75 | .69 | .26 | 3.56 | .002 | 1.48 | 21 |
|  |  | .80 | .79 | .22 | 6.47 | <.001 | 2.70 | >1000 |
| **5** | Baseline | .50 | .96 | .14 | 15.91 | <.001 | 6.63 | >1000 |
|  |  | .67 | .93 | .14 | 15.22 | <.001 | 6.35 | >1000 |
|  |  | .75 | .79 | .22 | 6.23 | <.001 | 2.60 | >1000 |
|  |  | .80 | .72 | .28 | 3.84 | .001 | 1.60 | 38 |
|  | Addition | .50 | .90 | .21 | 9.35 | <.001 | 3.90 | >1000 |
|  |  | .67 | .79 | .20 | 7.00 | <.001 | 2.92 | >1000 |
|  |  | .75 | .69 | .17 | 5.44 | <.001 | 2.27 | 1000 |
|  |  | .80 | .81 | .22 | 6.83 | <.001 | 2.85 | >1000 |
| Unknown-addend | | .50 | .85 | .23 | 7.47 | <.001 | 3.12 | >1000 |
|  |  | .67 | .74 | .23 | 5.17 | <.001 | 2.16 | 1000 |
|  |  | .75 | .70 | .19 | 4.98 | <.001 | 2.08 | 500 |
|  |  | .80 | .70 | .19 | 4.98 | <.001 | 2.08 | 500 |
| **6** | Baseline | .50 | .96 | .14 | 15.91 | <.001 | 6.63 | >1000 |
|  |  | .67 | .95 | .10 | 21.16 | <.001 | 8.82 | >1000 |
|  |  | .75 | .86 | .13 | 14.04 | <.001 | 5.86 | >1000 |
|  |  | .80 | .82 | .14 | 11.50 | <.001 | 4.80 | >1000 |
|  | Addition | .50 | .94 | .17 | 12.69 | <.001 | 5.29 | >1000 |
|  |  | .67 | .76 | .17 | 7.39 | <.001 | 3.08 | >1000 |
|  |  | .75 | .72 | .20 | 5.38 | <.001 | 2.24 | 1000 |
|  |  | .80 | .83 | .19 | 8.58 | <.001 | 3.58 | >1000 |
| Unknown-addend | | .50 | .91 | .19 | 9.35 | <.001 | 3.90 | >1000 |
|  |  | .67 | .77 | .22 | 6.03 | <.001 | 2.51 | >1000 |
|  |  | .75 | .67 | .18 | 4.65 | <.001 | 1.94 | 250 |
|  |  | .80 | .76 | .25 | 5.11 | <.001 | 2.13 | 1000 |

Table S4. Descriptive statistics, results of One-Sample t tests comparing to chance level (.5), and results of Bayes factor analyses for each comparison ratio in Baseline, Addition and Unknown-addend conditions in 4-, 5-, and 6-year-olds. Alpha criterion for significance was set to .017, correcting for multiple comparison within each age group and condition. Bayes factor indicates the odds of the alternative hypothesis that children correctly chose the larger quantity set above chance, over the null hypothesis that children’s judgement of the larger set was not different from chance.

# Investigating alternative response strategies

## Addition condition.

### Extreme of range strategy.

This is a strategy in which children’s judgment is based on the largest quantity they observed. For example, if *one* of the addends was larger than the comparison array, the summed total of the addends would also be larger than the comparison array; if children responded based on the size of the addend, they would still be responding correctly without summing over both addends. In our experiment, this strategy could only be used in one of the test trials (6+12 vs. 9), in which 64/72 children succeeded (binomial *p* < .001). After excluding this trial, children’s performance was still above chance (*t*(71) = 14.23, *p* < .001, *d* *=* 3.38, BF_10_ > 1000).

### Near/far strategy.

In this strategy, children may compare the larger addend with the comparison array, and then make an inference without actually computing the sum. That is, if the larger addend is close in quantity to the comparison array, children may infer that the summed total will be larger than the comparison array; if the larger addend is smaller than the comparison array, children may infer that the summed total will be smaller than the comparison array. To examine whether children adopted this strategy, we removed trials in which the larger addend was close in quantity to the comparison array (7 trials in which the difference between the larger addend array and the comparison array is between 2-5 items, e.g., 10 + 14 vs. 16) and trials in which the larger addend was far in quantity from the comparison array (6 trials in which the difference between the larger addend array and the comparison array was between 11-21 items, e.g., 12+15 vs. 36). For the remaining one trial, in which the ratio between the larger addend and the comparison array was not too large and not too small (5+7 vs. 16, where the difference between the larger addend and the comparison array is 9), if children were using the near/far strategy, they should not perform above chance on these trials. We found that 47/72 (65%) children succeeded in those trials (binomial *p* = .013), suggesting children were not using the near/far distance strategy.

### Scale one operand strategy.

The nature of summation operation requires children to hold an occluded quantity (addend 1) in working memory and to then manipulate that quantity (summing it with addend 2), which places further demands on working memory. To reduce working memory load, children may adopt a strategy of selecting one of the addends and simply doubling it (e.g., first operand X 2). Previous work has shown that 5- to 8-year-olds can perform a non-symbolic scaling operation (up or down; McCrink & Spelke, 2010, 2016; Qu, Szkudlarek, & Brannon, 2021), suggesting children in our study may be able to adopt such strategy. To examine whether children in our task used this strategy, we analyzed trials in which adopting such a strategy would lead to incorrect or chance-level responses (there were two such trials). Children succeed in both trials (first operand multiplies by 2: 13+7 vs. 25: 61/72 (85%) succeeded, binomial *p* < .001; either operand multiplies by 2: 6+4 vs. 8: 51/72 (71%) children succeeded, binomial *p* < .001).

## Unknown-addend condition.

### Extreme of range strategy.

In the Unknown-addend condition, this strategy would involve comparing the value of the final quantity (after the unknown addend was added) to the comparison quantity, rather than computing the value of the unknown addend. There was only one trial in which this strategy would reliably produce an above-chance response (4 + Gator = 14 vs. 20). Excluding this trial, children’s responses on the remaining trials were still significantly above chance (*t*(71) = 13.43, *p* < .001, *d* = 3.19, BF_10_ >1000).

### Near/far strategy.

In the Unknown-addend condition, this strategy involves using the difference between the final quantity (after the unknown addend is added) and the comparison quantity to infer whether the unknown addend was larger or smaller than the comparison quantity: if the final quantity is close to the comparison quantity, the unknown added should be smaller than the comparison; if the final quantity is much larger than the comparison quantity, the unknown addend is likely to be larger than the comparison. To examine whether children used this strategy, we analyzed trials where adopting such strategy would lead to chance or below chance performance (Trials 8, 10, 11, 13, and 14, see Supplement Table S6). We found that children were significantly above chance in those trials (*t*(71) = 6.85, *p* < .001, *d* = 1.63, BF_10_ > 1000).

### Scale final quantity strategy.

This strategy involves simply dividing the final quantity after the unknown addend is added in half, and comparing the scaled-down quantity to the comparison array. To examine whether children used this strategy, we analyzed the trials in which such strategy would result in incorrect or chance-level responses (Trials 8, 10, 12, 14; see Supplement Table S6). We found children’s performance was significantly above chance in those trials (*t*(71) = 3.22, *p* = .002, *d* = .76, BF_10_ = 10.99).

# Individual trial summary in the Addition condition

| Trial | Ratio | Gator Set1 | Gator Set2 | Gator total | Cheetah | Mean proportion correct |
| --- | --- | --- | --- | --- | --- | --- |
| Practice 1 | .50 | 4 | 6 | 10 | 20 | 0.81 |
| Practice 2 | .50 | 6 | 4 | 10 | 5 | 0.92 |
| Test 1 | .50 | 4 | 6 | 10 | 20 | 0.88 |
| Test 2 | .50 | 6 | 12 | 18 | 9 | 0.89 |
| Test 3 | .67 | 7 | 5 | 12 | 18 | 0.71 |
| Test 4 | .67 | 11 | 10 | 21 | 14 | 0.72 |
| Test 5 | .67 | 10 | 6 | 16 | 24 | 0.86 |
| Test 6 | .67 | 10 | 14 | 24 | 16 | 0.72 |
| Test 7 | .75 | 5 | 7 | 12 | 16 | 0.65 |
| Test 8 | .75 | 8 | 12 | 20 | 15 | 0.68 |
| Test 9 | .75 | 12 | 15 | 27 | 36 | 0.88 |
| Test 10 | .75 | 14 | 22 | 36 | 27 | 0.48 |
| Test 11 | .80 | 13 | 15 | 28 | 35 | 0.90 |
| Test 12 | .80 | 6 | 4 | 10 | 8 | 0.71 |
| Test 13 | .80 | 13 | 7 | 20 | 25 | 0.85 |
| Test 14 | .80 | 7 | 8 | 15 | 12 | 0.71 |

Table S5. Children’s mean proportion correct for each trial in the Addition condition. Note that it does not reflect the actual running trial number, as the trials were run based on two pre-determined pseudorandomized orders (see Table S2).

# Individual trial summary in the Unknown-addend condition

| Trial | Ratio | Starting pile | Ending pile | Gator | Cheetah | Mean proportion correct |
| --- | --- | --- | --- | --- | --- | --- |
| Practice 1 | .50 | 4 | 9 | 5 | 10 | 0.78 |
| Practice 2 | .50 | 6 | 26 | 20 | 10 | 0.93 |
| Practice 3 | .50 | 6 | 24 | 18 | 9 | 0.82 |
| Practice 4 | .50 | 14 | 20 | 6 | 12 | 0.46 |
| Test 1 | .50 | 4 | 14 | 10 | 20 | 0.94 |
| Test 2 | .50 | 6 | 24 | 18 | 9 | 0.82 |
| Test 3 | .67 | 7 | 19 | 12 | 18 | 0.79 |
| Test 4 | .67 | 11 | 32 | 21 | 14 | 0.71 |
| Test 5 | .67 | 10 | 26 | 16 | 24 | 0.86 |
| Test 6 | .67 | 10 | 34 | 24 | 16 | 0.63 |
| Test 7 | .75 | 5 | 17 | 12 | 16 | 0.85 |
| Test 8 | .75 | 8 | 28 | 20 | 15 | 0.61 |
| Test 9 | .75 | 12 | 39 | 27 | 36 | 0.78 |
| Test 10 | .75 | 14 | 50 | 36 | 27 | 0.50 |
| Test 11 | .80 | 13 | 41 | 28 | 35 | 0.86 |
| Test 12 | .80 | 6 | 16 | 10 | 8 | 0.76 |
| Test 13 | .80 | 13 | 33 | 20 | 25 | 0.79 |
| Test 14 | .80 | 7 | 22 | 15 | 12 | 0.58 |

Table S6. Children’s mean proportion correct for each trial in the Unknown-addend condition. Note that it does not reflect the actual running trial number, as the trials were run based on two pre-determined pseudorandomized orders (see Table S3).
